# Advances in adoptive cell therapies in small cell lung cancer

**DOI:** 10.37349/etat.2025.1002302

**Published:** 2025-03-26

**Authors:** Eljie Isaak Bragasin, Justin Cheng, Lauren Ford, Darin Poei, Sana Ali, Robert Hsu

**Affiliations:** Alma Mater Studiorum Università di Bologna, Italy; ^1^Keck School of Medicine, University of Southern California, Los Angeles, CA 90033, USA; ^2^Department of Internal Medicine, University of Southern California, Los Angeles, CA 90033, USA; ^3^Department of Medicine, Division of Medical Oncology, University of Southern California Norris Comprehensive Cancer Center, Los Angeles, CA 90033, USA

**Keywords:** Small cell lung cancer, antibody drug conjugates, bispecific T-cell engagers, CAR-T cell therapies

## Abstract

Small cell lung cancer (SCLC) is an aggressive tumor characterized by early metastasis and resistance to treatment, making it a prime target for therapeutic investigation. The current standard of care for frontline treatment involves a combination of chemotherapeutic agents and immune checkpoint inhibitors (ICIs), though durability of response remains limited. The genetic heterogeneity of SCLC also complicates the development of new therapeutic options. Adoptive cell therapies show promise by targeting specific mutations in order to increase efficacy and minimize toxicity. There has been significant investigation in three therapeutic classes for application towards SCLC: antibody drug conjugates (ADCs), bispecific T-cell engagers (BiTEs), and chimeric antigen receptor (CAR)-T cell therapies. This review summarizes the recent advances and challenges in the development of adoptive cell therapies. Genetic targets such as delta-like ligand 3 (DLL3), trophoblast cell surface antigen 2 (Trop2), B7-H3 (CD276), gangliosides disialoganglioside GD2 (GD2) and ganglioside GM2 (GM2) have been found to be expressed in SCLC, which makes them prime targets for therapy development. While investigated therapies such as rovalpituzumab tesirine (Rova-T) have failed, several insights from these trials have led to the development of compelling new agents such as sacituzumab govitecan (SG), ifinatamab deruxtecan (I-DXd), tarlatamab, and DLL3-targeted CAR-T cells. Advancing development of molecular testing and improving targeted approaches remain integral to pushing forward the progress of adoptive cell therapies in SCLC.

## Introduction

Lung cancer accounts for 1 in every 5 cancer deaths and is currently the leading cause of cancer-related mortality [1]. Small cell lung cancer (SCLC) makes up 14% of lung cancers and carries a high mortality rate relative to other solid tumors, with a 5-year survival rate of 7% [2]. This cancer’s aggressiveness is characterized by its high mitotic rate, early metastatic spread, and resistance to treatment. Recent advancements have shed light on the cell of origin and molecular classification of SCLC. SCLC has long been considered to be the result of near-universal loss of TP53 and RB1. However, multi-omic approaches to SCLC profiling have now enhanced our understanding of the tumor highlighted by a model of four distinct subtypes characterized by the relative expression of key transcriptional factors: achaete-scute homolog 1 (*ASCL1*), neurogenic differentiation factor 1 (*NeuroD1*), and POU class 2 homeobox 3 (*POU2F3*), corresponding to subtypes SCLC-A (*ASCL1* dominant), SCLC-N (*NeuroD1* dominant), and SCLC-P (*POU2F3* dominant), respectively. A fourth subtype, SCLC-I, represents an inflamed subtype with low expression of *ASCL1*, *NeuroD1*, and *POU2F3* [3]. Notably, SCLC-P is now believed to originate from pulmonary tuft cells, given its close transcriptional profile [4]. Ongoing research aims to clarify the clinical significance of molecular subtype classification, as well as the distinct developmental origins and progression pathways of each subtype.

At diagnosis, nearly 60% of patients present with extensive-stage disease, with common sites of metastasis including the brain, liver, adrenal glands, and bone. Despite its initial sensitivity to chemoradiation, responses are short-lived with most patients relapsing within 6 months after completion of initial treatment [5]. In the past thirty years, there has been little progress in finding new systemic treatments for SCLC. For decades, platinum-based chemotherapies, such as cisplatin or carboplatin, have been the standard-of-care treatment [6]. Immune checkpoint inhibitors (ICIs) initially showed significant activity in melanoma and non-small cell lung cancer (NSCLC) [7, 8]. As such, their use in SCLC was highly anticipated [9]. The ICIs used in SCLC target the programmed cell death protein-1 (PD-1)/programmed death-ligand 1 (PD-L1) axis and are applied either as single agents or in combination with chemotherapy [6, 10]. Atezolizumab, an anti-PD-L1 monoclonal antibody (mAb), was the first ICI approved for extensive-stage SCLC (ES-SCLC) in combination with chemotherapy agents carboplatin and etoposide based on data from the IMpower133 trial (NCT02763579) [11], which found a median overall survival (OS) of 12.3 months [95% confidence interval (CI): 10.8–15.8] and median progression-free survival (PFS) of 5.2 months (95% CI: 4.4–5.6). Data from the IMbrella A extension study demonstrated a five-year OS of 12% for atezolizumab plus carboplatin and etoposide compared to the historical 2% five-year OS of chemotherapy alone [12]. Similarly, the CASPIAN trial (NCT03043872) provided promising results for another anti-PD-L1 mAb, durvalumab [13]. Patients who received durvalumab with etoposide plus cisplatin or carboplatin (EP) had an improved median OS of 12.9 months (95% CI: 11.3–14.7) compared to 10.5 months (95% CI: 9.3–11.2) for those who received EP only. PFS rates for the durvalumab with EP group were 85.2% (95% CI: 65.2–94.2) PFS at 12 months and 81.5% (95% CI: 61.1–91.8) at 24 months. In an updated analysis, 3-year OS for the durvalumab plus EP group was 17.6% compared to 5.8% in the EP group [13].

Though the approvals of these ICIs were the first advances in SCLC treatment in decades, the data from the IMpower133 and CASPIAN trials demonstrated only 2–3 months of improvement in OS and long-term efficacy in only 10–15% of patients [11, 13]. While the high mutation burden of SCLC allows for its immunotherapeutic targeting, it may also explain treatment resistance and poor long-term outcomes of the disease [14, 15]. ​​​​Any minimal residual disease (MRD) in SCLC can quickly develop resistance to first-line agents and grow back more aggressively. A better understanding of the molecular mechanisms of resistance in SCLC, especially of MRD cells, could potentially enhance the efficacy of its immunotherapies. In terms of treatment options for relapsed SCLC, there are a plethora of challenges, including limited subsequent available therapies and poor sustainable efficacy. The FDA recently withdrew approval for certain immunotherapies, including nivolumab and pembrolizumab, for relapsed SCLC due to a lack of OS benefit ​[16–18]. The optimal length of subsequent therapy in relapsed SCLC is not well studied and current treatment recommendations are determined on a case-by-case basis [19]. The sparse research and understanding of SCLC treatments is likely due to the inability to easily biopsy and test for predictive biomarkers. In years past, it had been difficult to obtain adequate tissue material for analysis. Recent developments in liquid biopsies have shown promise due to their minimally invasive nature and ability to monitor disease [20]. In the past, the inability to assess predictive biomarkers hindered targeted therapy development, but liquid biopsy and its test results intend to transform disease management through a personalized approach [21]. There is extensive exploration in genetic testing with the hope of further classifying SCLC genes [22]. This includes identifying lung cancer risk factors, such as loci variants linked to increased risk of developing SCLC [23], and tumor profiling in active SCLC cases. 90% of SCLC tumors harbor biallelic inactivation of both tumor suppressor genes *RB1* and *TP53* [23, 24]. Despite this common loss, SCLC is characterized by molecular complexity with poorly understood genomic patterns to resistance [23, 25]. Next steps include categorizing SCLC-specific phenotypes, with studies showing that amplification patterns of MYC, MYCL, and MYCN may assist in further categorization of this cancer [23, 26, 27]. These advancements in the testing of tissue and expression patterns of SCLC-related genes have allowed for increasingly individualized care for patients with SCLC [22, 28].

**Figure 1:**
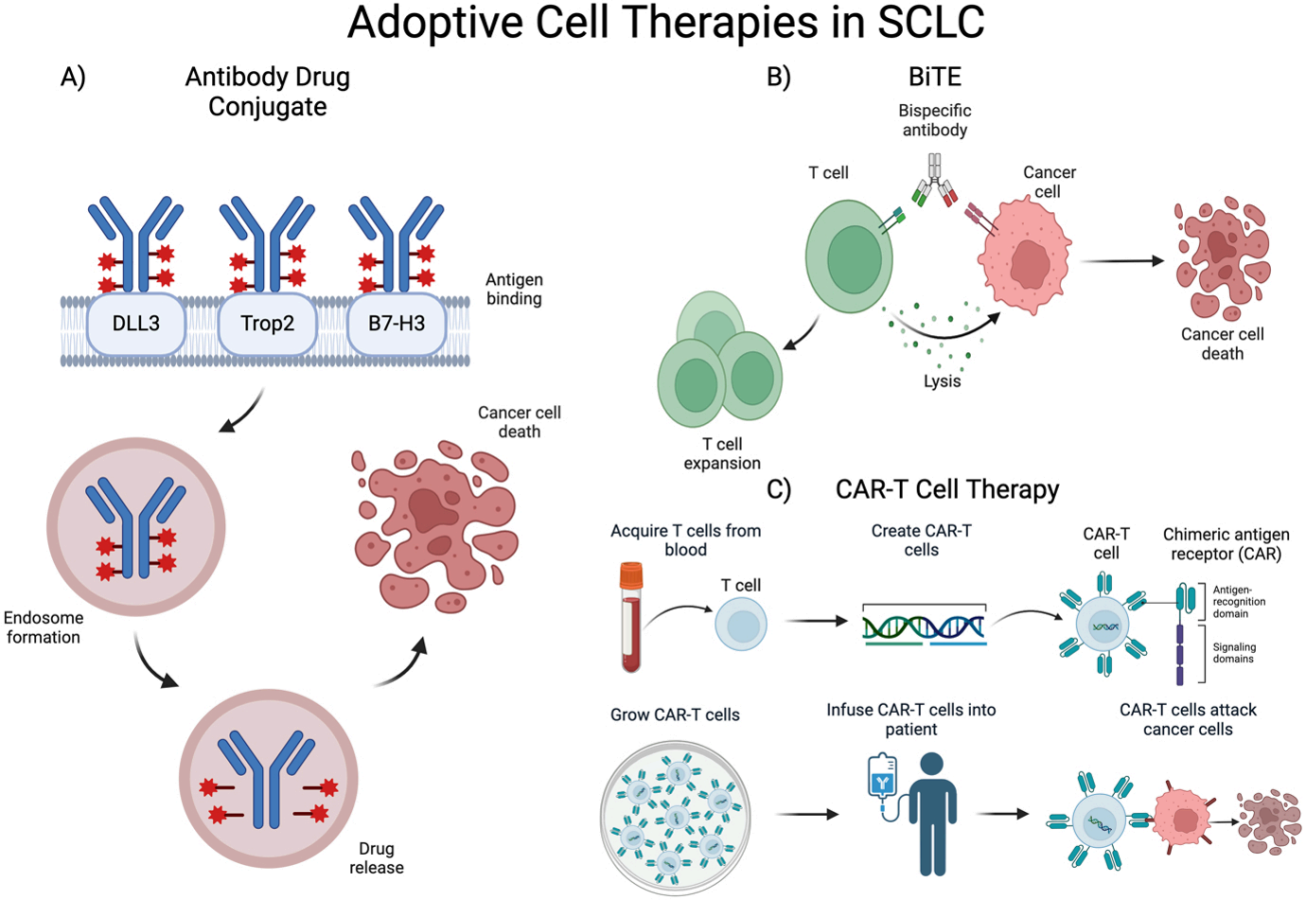
**Mechanism of action of adoptive cell therapies in SCLC**. (**A**) Mechanism of action of ADC: mAb binds gene target, ADC is then phagocytosed and stored in an endosome, cytotoxic drug is released within cells expressing gene target; (**B**) mechanism of action of BiTE therapy: dual mAb against tumor-associated antigen and CD3 recruit T cells to tumor cells leading to cell lysis and clonal expansion of T cells; (**C**) mechanism of action of CAR-T cell therapy: T cells are collected and modified to express a chimeric antigen receptor (CAR), CAR-T cells are then expanded in culture and infused to the patient, CAR-T cells then target cells expressing the targeted surface protein [38]. SCLC: small cell lung cancer; DLL3: delta-like ligand 3; Trop2: trophoblast cell surface antigen 2; BiTE: bispecific T-cell engager; ADC: antibody drug conjugate; mAb: monoclonal antibody. Created in BioRender. Poei, D. (2025) https://BioRender.com/n76d352

Though treatment options for SCLC have been limited with few improvements in life expectancy and PFS, discovery of gene targets in SCLC has contributed to a rapid expansion of studies of targeted treatments. Specifically, there are three classes of systemic therapy being investigated in SCLC: antibody drug conjugates (ADCs), bispecific T-cell engagers (BiTEs), and chimeric antigen receptor (CAR)-T cell therapies (Figure 1). ADCs were first developed in animal models in the 1960s with the first human clinical trials in the 1980s ​[29]. Initial trials were hindered by premature drug release [29, 30], but next-generation ADCs show significant activity against treatment-resistant cancers [31]. In ADCs, a mAb binds to a tumor cell’s target antigen, allowing for lysosomal fusion, cytotoxic release, and apoptosis of cells [29, 31]. BiTEs are bispecific mAbs that bind CD3 on T cells and a tumor antigen to activate T-cell mediated tumor lysis [32]. Developed in the 1980s, BiTEs have unique antigen-binding sites in each arm, along with an Fc domain to enhance T-cell activation [32, 33]. CAR-T cell therapy development began in the 1990s with engineered T-cells showing anti-cancer activity in animals [34, 35]. The FDA approved the first CAR-T therapy in 2017 after successful clinical trials [34, 36]. This immunotherapy uses the patient’s T-cells, modifying them to better express a synthetic receptor that can recognize and attack cancer cells. These cells are then infused back into the patient where they bind tumor antigens and, once activated, release cytotoxic chemicals to kill cancer cells [37]. Below, we discuss the results from key studies of these three classes of targeted therapies, ADCs, BiTEs, and CAR-T cell therapies, and how they are being investigated in SCLC.

## ADCs in SCLC

**Figure 2:**
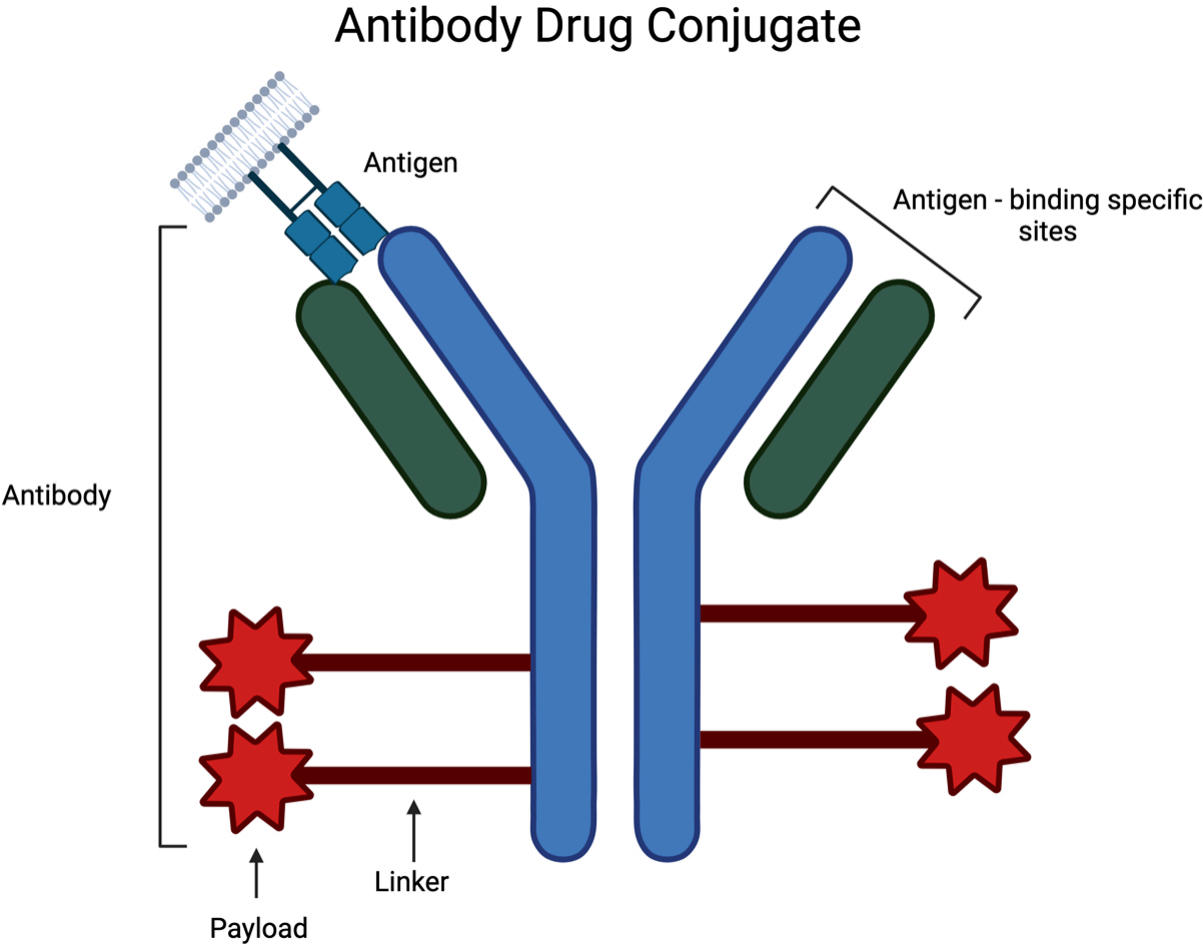
**Components of antibody drug conjugate including recombinant monoclonal antibody with antigen binding sites, synthetic linker, and cytotoxic chemical payload** [38]. Created in BioRender. Poei, D. (2025) https://BioRender.com/d11b497

**Figure 3:**
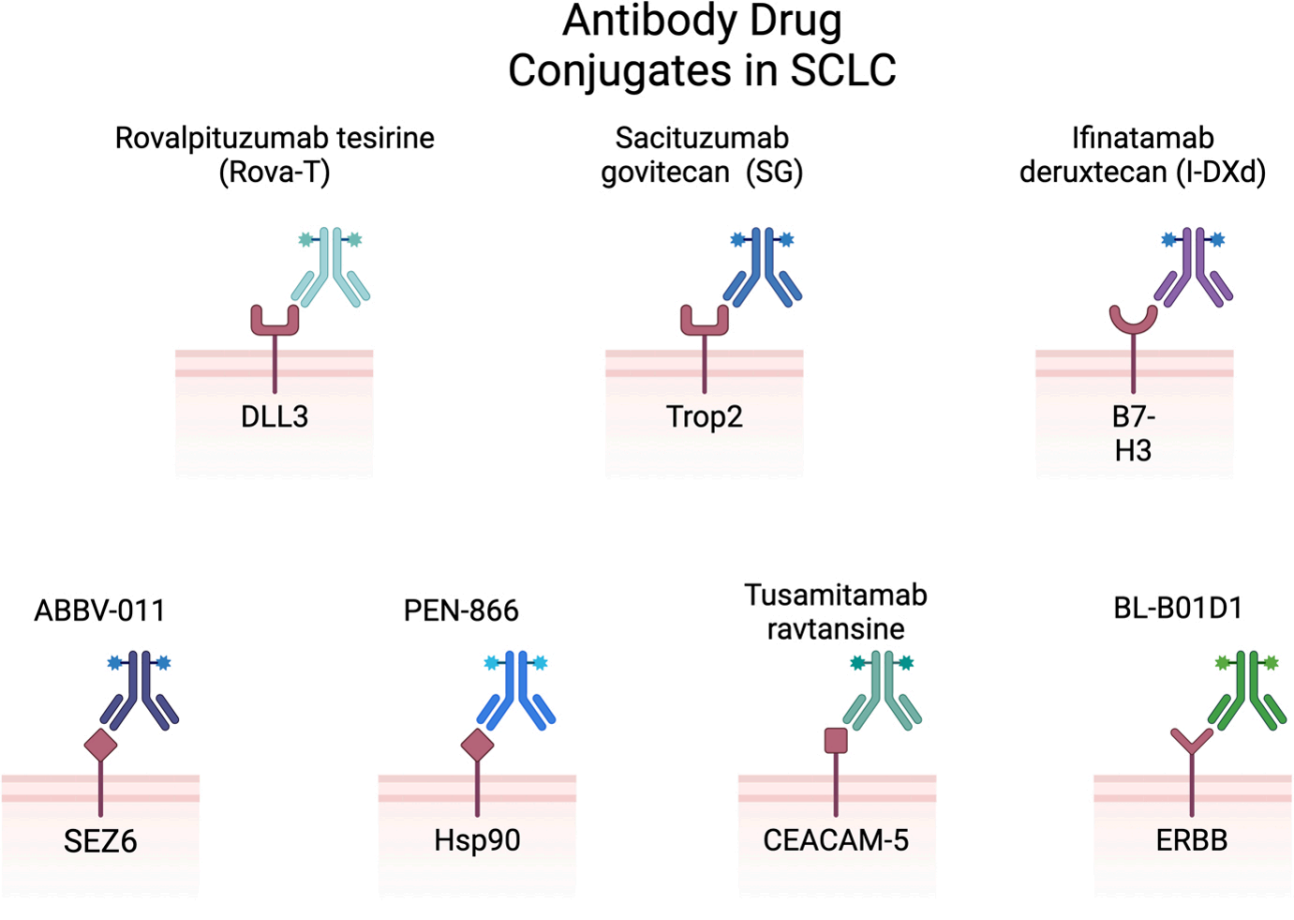
**ADCs in SCLC include rovalpituzumab tesirine (anti-DLL3), sacituzumab govitecan (anti-Trop2), infinatamab deruxtecan (anti-B7-H3), ABBV-011 (anti-SEZ6), PEN-866 (anti-Hsp90), tusamitamab ravtansine (anti-CEACAM5), BL-B01D1 (anti-ERBB)** [38]. SCLC: small cell lung cancer; DLL3: delta-like ligand 3; Trop2: trophoblast cell surface antigen 2; SEZ6: seizure-related homolog 6; Hsp90: heat shock protein 90; CEACAM5: carcinoembryonic antigen-related cell adhesion molecule 5; ADC: antibody drug conjugate. Created in BioRender. Poei, D. (2025) https://BioRender.com/y56d720

ADCs are a rapidly growing class of oncology therapeutics. ADCs are composed of recombinant mAbs bound to cytotoxic chemicals via synthetic linkers (Figure 2) [39]. This combination leads to highly selective administration of cytotoxic payload [39]. ADC design involves the identification and optimization of each of these components. Target antigen is often selected through genetic studies that identify antigens that are highly expressed on tumor cells and minimally expressed or accessible in normal tissue to limit off-tumor toxicity [40]. The cytotoxic payloads of ADCs are often selected to be easily attachable to the chosen antibody, highly toxic, and hydrophilic [39]. The synthetic linkers used in ADCs are also optimized to increase stability in circulation yet allow for appropriate release of payload inside the target cell. Many advancements in ADC design have occurred due to the years of studies in both hematologic and solid tumor malignancies. There have been 14 completed trials including patients with SCLC investigating 8 different ADCs (Figure 3 and Table 1): rovalpituzumab tesirine (Rova-T), sacituzumab govitecan (SG), ifinatamab deruxtecan (I-DXd), HS-20093, ABBV-011, ABBV-706, BL-B01D1, and tusamitamab ravtansine. There are 20 active trials including patients with SCLC investigating 9 ADCs (Table 2): FZ-AD005, SG, sacituzumab tirumotecan, datopotamab deruxtecan (Dato-DXd), I-DXd, HS-20093, ABBV-706, BL-B01D1, and tusamitamab ravtansine. We discuss the outcomes and progress of these trials below.

**Table 1:** Key trials of ADCs in SCLC

**Agent**	**Target**	**Trial identifier**	**Phase**	**ORR**	**OS**	**PFS**	**Grade 3 or > TRAEs**
Rovalpituzumab tesirine	DLL3	NCT01901653	I	18%		2.8 mo (95% CI: 2.5–4.0)	38%
Rovalpituzumab tesirine	DLL3	TRINITY (NCT02674568)	II	12.4%	5.6 mo (95% CI: 4.9–6.1)	3.5 mo (95% CI: 3.0–3.9)	63%
Rovalpituzumab tesirine	DLL3	MERU (NCT03033511)	III		8.5 mo (95% CI: 7.3–10.2)	4.0 mo (95% CI: 3.2–4.1)	59%
Rovalpituzumab tesirine	DLL3	TAHOE (NCT03061812)	III		6.3 mo (95% CI: 5.6–7.3)	3.0 mo (95% CI: 2.9–3.6)	64%
Rovalpituzumab tesirine + ipilimumab & nivolumab	DLL3	NCT03026166	I/II	36.4% (95% CI: 10.9–69.2)	11.0 mo (95% CI: 2.3–17.0)	4.1 mo (95% CI: 1.3–6.0)	100%
Rovalpituzumab tesirine + nivolumab	DLL3	NCT03026166	I/II	27.6% (95% CI: 12.7–47.2)	7.4 mo (95% CI: 5.0–9.1)	4.8 mo (95% CI: 3.2–5.3)	87%
Sacituzumab govitecan	Trop2	IMMU-132-01 (NCT01631552)	I/II	17.70%	7.1 mo (95% CI: 5.6–8.1)	3.7 mo (95% CI: 2.1–4.8)	59.6%
Sacituzumab govitecan	Trop2	TROPiCS-03 (NCT03964727)	II	29% (95% CI: 8–58)			46%
Ifinatamab deruxtecan	B7-H3	NCT04145622	I/II	52%	9.9 mo	5.8 mo	
HS-20093	B7-H3	ARTEMIS-001 (NCT05276609)	I	8 mg/kg dose: 58.1%		5.6 mo	
ABBV-011	SEZ6	NCT03639194	I	25%		3.5 mo	47.5%
ABBV-706	SEZ6	NCT05599984	I	21%			57%
BL-B01D1	EGFR-HER3	NCT05194982	I	34%		5.7 mo	71%
Tusamitamab ravtansine	CEACAM5	NCT02187848	I				14.3% (Q2W-LD), 13.3% (Q3W)

ADCs: antibody drug conjugates; SCLC: small cell lung cancer; DLL3: delta-like ligand 3; Trop2: trophoblast cell surface antigen 2; SEZ6: seizure-related homolog 6; EGFR: epidermal growth factor receptor; CEACAM5: carcinoembryonic antigen-related cell adhesion molecule 5; CI: confidence interval; Q2W: every 2 weeks; ORR: objective response rate; OS: overall survival; PFS: progression-free survival; TRAEs: treatment-related adverse events; mo: month

**Table 2:** Active trials of ADCs in SCLC

**Agent**	**Phase**	**Status**	**Trial identifier**	**Indications**	**Sponsor**
FZ-AD005	I	Recruiting	NCT06424665	Advanced solid tumors	Shanghai Fudan-Zhangjiang Bio-Pharmaceutical Co.
Sacituzumab govitecan	I/II	Recruiting	NCT04826341	SCLC, extra-pulmonary small cell neuroendocrine cancer & homologous recombination-deficient cancers resistant to PARP inhibitors	National Cancer Institute
Sacituzumab govitecan	II	Active, not recruiting	NCT03964727	Advanced solid tumors	Gilead Sciences
Sacituzumab govitecan	II	Not yet recruiting	NCT06667167	ES-SCLC	Nir Peled
Sacituzumab tirumotecan	I/II	Recruiting	NCT04152499	Advanced solid tumors	Klus Pharma Inc.
Datopotamab deruxtecan	I	Recruiting	NCT03401385	Advanced solid tumors	Daiichi Sankyo
Ifinatamab deruxtecan	I/II	Recruiting	NCT04145622	Advanced solid tumors	Daiichi Sankyo
Ifinatamab deruxtecan	Ib/II	Recruiting	NCT06362252	ES-SCLC	Daiichi Sankyo
Ifinatamab deruxtecan	II	Active, not recruiting	NCT05280470	ES-SCLC	Daiichi Sankyo
Ifinatamab deruxtecan	III	Recruiting	NCT06203210	Relapsed SCLC	Daiichi Sankyo
HS-20093	I	Recruiting	NCT05276609	Advanced solid tumors	Hansoh BioMedical R&D Company
HS-20093	III	Recruiting	NCT06498479	Relapsed SCLC	Hansoh BioMedical R&D Company
HS-20093	III	Not yet recruiting	NCT06526624	Limited-stage SCLC	Hansoh BioMedical R&D Company
ABBV-706	I	Recruiting	NCT05599984	Advanced solid tumors	AbbVie
BL-B01D1	Ia	Recruiting	NCT05194982	Locally advanced or metastatic solid tumor	Sichuan Baili Pharmaceutical Co., Ltd.
BL-B01D1	II	Recruiting	NCT05924841	ES-SCLC	Sichuan Baili Pharmaceutical Co., Ltd.
BL-B01D1	II	Recruiting	NCT06437509	ES-SCLC	Sichuan Baili Pharmaceutical Co., Ltd.
BL-B01D1	III	Recruiting	NCT06500026	Relapsed SCLC	Sichuan Baili Pharmaceutical Co., Ltd.
BL-B01D1	I/IIa	Recruiting	NCT06618287	Advanced solid tumors	Bristol-Myers Squibb
Tusamitamab ravtansine	I	Active, not recruiting	NCT02187848	Advanced solid tumors	Sanofi

ADCs: antibody drug conjugates; SCLC: small cell lung cancer; ES-SCLC: extensive-stage SCLC

### Delta-like ligand 3 (DLL3)

Delta-like ligand 3 (DLL3), an atypical Notch ligand and novel biomarker of tumor-initiating cells has been identified as a potential target in SCLC [41]. DLL3 has been shown to be highly expressed across SCLC disease stages and remains stable despite treatment making it a good target for oncologic therapies [42].

Rova-T was the first DLL3-targeted ADC investigated in SCLC and consisted of the humanized DLL3-specific IgG1 mAb SC16, a protease-cleavable linker, and the DNA crosslinker SC-DR002. A phase I trial (NCT01901653) in recurrent SCLC demonstrated the safety profile of Rova-T, as 38% of study participants experienced grade 3 or worse treatment-related adverse events (TRAEs) with thrombocytopenia (11%), pleural effusion (8%), and increased lipase (7%) being the most common [41]. Serious TRAEs were observed in 43% of patients, the most common of which were pleural effusion (19%) and pericardial effusion (7%). Rova-T demonstrated encouraging antitumor activity as a single agent with confirmed objective response (OR) in 18% of patients and 38% of those with high DLL3 expression (expression in at least 50% of tumor cells). Median PFS was 2.8 months (95% CI: 2.5–4.0) for all patients and 4.3 months (95% CI: 2.8–5.6) for those with high DLL3 expression.

Rova-T was subsequently studied as a therapy for third-line and beyond in patients with DLL3-expressing relapsed/refractory SCLC in the TRINITY trial (NCT02674568) [43]. This phase II trial utilized a rabbit antibody immunohistochemistry (IHC) assay to stratify study participants based on the percent expression of DLL3 found in tumor samples with at least 25% expression defined as DLL3-positive and at least 75% expression defined as DLL3-high. Response data demonstrated only modest clinical activity with OR in 14.3% of DLL3-high patients, 13.2% in DLL3-positive patients, and 12.4% of all patients studied. OS was 5.7 months (95% CI: 4.9–6.7) in DLL3-high patients, 5.8 months (95% CI: 5.1–6.7) in DLL3-positive patients, and 5.6 months (95% CI: 4.9–6.1) for all patients. PFS was a secondary measured endpoint and was found to be 3.8 months (95% CI: 3.2–4.1) in DLL3-high patients, 3.8 months (95% CI: 3.2–4.0) in DLL3-positive patients, and 3.5 months (95% CI: 3.0–3.9) for all patients. Grade 3 and above TRAEs were seen in 63% of study participants with the most common being thrombocytopenia (13%), photosensitivity reaction (7%), pleural effusion (6%), and anemia (6%).

Two phase III studies evaluating Rova-T as a single agent were conducted after the TRINITY trial. The MERU trial (NCT03033511) compared Rova-T to placebo as maintenance therapy for patients with ES-SCLC, previously treated with first-line platinum-based chemotherapy [44]. This study showed a lack of OS benefit for Rova-T as a maintenance therapy for ES-SCLC as median OS for Rova-T was 8.5 months (95% CI: 7.3–10.2) vs. 9.8 months (95% CI: 8.4–10.9) for placebo, but PFS was increased for the Rova-T arm [4.0 months (95% CI: 3.2–4.1)] compared to the placebo arm [1.4 months (95% CI: 1.4–1.5)]. Grade 3 or higher TRAEs were recorded in 59% of those who received Rova-T with the most common being thrombocytopenia (9%), pleural effusion (4%), and photosensitivity reaction (4%). 30% of patients within the placebo group experienced grade 3 or higher TRAEs with thrombocytopenia (2%) and increased aspartate aminotransferase (2%) as the two most common.

The TAHOE trial (NCT03061812) studied Rova-T as second-line therapy for patients with DLL3-high SCLC [45]. Rova-T was compared against the current standard second-line treatment topotecan and was found to have an inferior OS of 6.3 months (95% CI: 5.6–7.3) compared to the 8.6 months (95% CI: 7.7–10.1) with topotecan. Median PFS was similarly decreased for the Rova-T arm at 3.0 months (95% CI: 2.9–3.6) vs. 4.3 months (95% CI: 3.8–5.4) for the topotecan arm. With regards to safety, 64% of patients who received Rova-T experienced grade 3 or higher TRAEs compared to 88% in the topotecan group. The most common grade 3 or higher TRAEs for Rova-T included malignant neoplasm progression (11%), thrombocytopenia (9%), dyspnea (8%), and anemia (7%). 56% of the Rova-T arm experienced serious TRAEs compared to 57% in the topotecan arm. Malignant neoplasm progression (10%), pneumonia (7%), pleural effusion (6%), and dyspnea (6%) were the most common serious TRAEs in the Rova-T arm compared to malignant neoplasm progression (13%), febrile neutropenia (9%), and thrombocytopenia (8%) in the topotecan arm.

In addition to these single agent studies, Rova-T has also been investigated as a combination therapy. In a phase I/II trial (NCT03026166), Rova-T in combination with nivolumab with and without ipilimumab was studied in patients with previously treated ES-SCLC with hopes of improvements in the individual clinical benefit demonstrated by these therapies [46]. Study participants were divided into two groups: cohort 1 received Rova-T with nivolumab and cohort 2 received Rova-T with nivolumab and ipilimumab. The studied combinations in this phase I/II study were not well tolerated with 87% of cohort 1 and 100% of cohort 2 experiencing grade 3 or higher TRAEs. The most common grade 3 or greater TEAEs for cohort 1 were anemia (17%), thrombocytopenia (13%), hyponatremia (13%), and pericardial effusion, pneumonitis, pleural effusion, hypertension, and hypophosphatemia (10% each). The most common grade 3 or greater TRAEs for cohort 2 were anemia (33%), thrombocytopenia (25%), fatigue (17%), dehydration (17%), and hyponatremia (17%).  Serious TRAEs were observed in 77% of cohort 1 and 75% of cohort 2. Collectively, 76% of participants experienced serious TRAEs with pleural effusion (19%) and pneumonitis (10%) being the most common. Objective response rate (ORR) was 27.6% (95% CI: 12.7–47.2) for cohort 1, 36.4% (95% CI: 10.9–69.2) for cohort 2, and 30.0% (95% CI: 16.6–46.5) overall. Median PFS was 4.8 months (95% CI: 3.2–5.3) for cohort 1, 4.1 months (95% CI: 1.3–6.0) for cohort 2, and 4.2 months (95% CI: 3.2–5.3) overall. Median OS was 7.4 months (95% CI: 5.0–9.1) for cohort 1, 11.0 months (95% CI: 2.3–17.0) for cohort 2, and 7.4 months (95% CI: 5.0–10.1) for all evaluable study participants. Overall, the lack of tolerability at the evaluated dose levels and administration schedules led to the discontinuation of study enrollment. Although Rova-T showed promise in early clinical trials, the lack of survival benefit in later studies terminated further development of Rova-T (Table 3).

**Table 3:** Terminated and withdrawn trials of ADCs

**Agent**	**Trial identifier**	**Status**	**Reason for termination/withdrawal**
Rovalpituzumab tesirine	NCT02709889	Terminated	Strategic considerations
Rovalpituzumab tesirine	NCT02819999	Terminated	Strategic considerations
Rovalpituzumab tesirine	NCT03026166	Terminated	Enrollment was stopped after the dose-limiting toxicity (DLT) evaluation phase of cohort 2
Rovalpituzumab tesirine	NCT03033511	Terminated	Independent Data Monitoring Committee recommendation
Rovalpituzumab tesirine	NCT03334487	Withdrawn	Strategic considerations
HS-20093	NCT06052423	Withdrawn	Research and development strategy adjustment
Tusamitamab ravtansine	NCT02187848	Terminated	Sponsor decision

ADCs: antibody drug conjugates

Though Rova-T was abandoned, its early successes inspired further exploration in ADCs. Other anti-DLL3 ADCs are still under investigation including FZ-AD005, a novel anti-DLL3 ADC consisting of a novel anti-DLL3 antibody FZ-A038 and a valine-alanine (Val-Ala) dipeptide linker to conjugate DXd [47]. FZ-AD005 was demonstrated to have promising antitumor activity in in vitro studies with half-maximal inhibitory concentration (IC_50_) values from 0.152 nM to 4.295 nM. In vivo studies were conducted in one patient-derived xenograft (PDX) model (LU5236) and two SCLC cell line-derived xenograft (CDX) models (NCI-H889 & NCI-H82). The tumor growth inhibition (TGI) of FZ-AD005 in the PDX model ranged from 98–100% at several tested doses while the TGI ranged from 86% to 97% in the CDX models. Lurbinectedin was used as a comparison for the NCI-H82 model and only achieved a TGI of 23.48%. A phase I study (NCT06424665) of FZ-AD005 in advanced solid tumors is currently enrolling at the time of this review.

### Trophoblast cell surface antigen 2 (Trop2)

Trophoblast cell surface antigen 2 (Trop2) is a transmembrane glycoprotein first described as a cell surface marker of trophoblast cells which is highly expressed in various cancers including SCLC. Despite its expression in normal tissue, animal and human studies have demonstrated an acceptable safety profile with minimal off-target effects with anti-Trop2 targeted therapies [48–50]. Trop2 targeted ADCs have therefore been a therapy of interest.

SG is a Trop2-directed ADC that has demonstrated efficacy in other solid tumors including breast, GI, and gynecologic tumors. SG consists of an anti-Trop2 IgG antibody with a CL2A linker, and an SN-38 cytotoxic payload [51]. CL2A is designed to be stable in serum and cleavable in the low pH of the tumor microenvironment (TME) while SN-38 is a topoisomerase I inhibitor and the active metabolite of irinotecan. The phase I/II IMMU-132-01 basket trial (NCT01631552) of SG monotherapy in patients with relapsed or refractory epithelial cancers demonstrated manageable toxicity and therapeutic activity [52]. Grade 3 or greater TRAEs were recorded in 59.6% of patients with neutropenia (42.4%), anemia (10.3%), and diarrhea (7.9%) being the most common. Serious TRAEs occurred in 15.2% of patients, the most common being febrile neutropenia (4%) and diarrhea (2.8%). Overall, 8.3% of participants permanently discontinued treatment due to the associated AEs. SG had a 17.7% ORR in the SCLC cohort with 11 of 62 patients demonstrating partial response. No complete responses were noted. Median OS in SCLC patients was 7.1 months (95% CI: 5.6–8.1) and PFS was 3.7 months (95% CI: 2.1–4.8).

Preliminary data from the phase II TROPiCS-03 basket trial (NCT03964727) demonstrated the effectiveness of SG as second-line therapy in ES-SCLC [53]. 14 patients were included in the efficacy analysis which showed an ORR of 29% (95% CI: 8–58), all of which were partial responses. 26 patients were included in the safety analysis and grade 3 or greater TRAEs were observed in 46% though there were no deaths or discontinuation of therapy related to TRAEs.

SG has also been studied in combination with berzosertib, a DNA damage response inhibitor, in a phase I/II trial (NCT04826341). Preliminary data demonstrated tolerability and tumor regression in patients with advanced solid tumors who progressed on prior therapy [54]. This combination therapy was investigated in 12 patients in which the most common TRAEs were lymphopenia (41.7%), neutropenia (25%), and leukopenia (16.7%). Two study participants had partial response, one with neuroendocrine prostate cancer (NEPC) transformed from high-risk prostate adenocarcinoma and one with SCLC transformed from epidermal growth factor receptor (*EGFR*)-mutated NSCLC. Both patients had greater than 40% decrease in tumor dimension from baseline measurements. A phase II trial (NCT06667167) of SG with pembrolizumab as maintenance therapy for ES-SCLC has been announced though patient recruitment has yet to begin.

Through subsequent studies, SG received FDA approval for use in three treatment-resistant malignancies: HR-positive/HER2-negative breast cancer, triple-negative breast cancer (TNBC), and urothelial cancer. There are no phase III trials of SG monotherapy or in combination involving patients with SCLC to date. Several anti-Trop2 ADCs are under investigation in SCLC, including sacituzumab tirumotecan (SKB264/MK-2870). When compared to SG in preclinical trials, sacituzumab tirumotecan was found to have extended half-life, improved targeting, and increased antitumor activity [55]. A phase I/II trial (NCT04152499) of sacituzumab tirumotecan in patients with advanced solid tumors is currently enrolling at the time of this review with promising preliminary results in the gastric or gastroesophageal junction (GEJ), NSCLC, and metastatic TNBC cohorts [56–58]. Results from the SCLC cohort have yet to be released. Dato-DXd is another anti-Trop2 ADC that is being investigated in SCLC. The TROPION-PanTumor01 (NCT03401385) trial of Dato-DXd is set to accept patients with SCLC in the dose expansion stage of the trial given its demonstrated safety, tolerability, and efficacy in NSCLC [59].

### B7-H3 (CD276)

B7-H3 is part of the B7 family of ligands, a group of cell surface proteins that have similar immune-modulating properties and includes the well-studied PD-L1 ligand. It is highly expressed in various malignancies and has been associated with worse outcomes [60]. In a study comparing B7 family ligand expression in SCLC samples, B7-H3 was found to be the most expressed in this group with expression in 64.9% of the SCLC samples tested compared to 7.3% for PD-L1 expression, demonstrating the value of investigating B7-H3 as a therapeutic target for SCLC [61].

I-DXd is a novel B7-H3-targeting ADC with a deruxtecan payload. In a phase I/II trial (NCT04145622) in patients with advanced solid tumors unselected for B7-H3 expression, I-DXd demonstrated promising OS and manageable safety profile in SCLC, castration-resistant prostate cancer, and esophageal squamous cell carcinoma [62]. The SCLC subgroup of this trial had an ORR of 52%, median PFS of 5.8 months, and median OS of 9.9 months.

The phase II DS7300-127 trial (NCT05280470) in patients with ES-SCLC investigating the optimum dose and efficacy of I-DXd is pending final results. The phase III IDeate-Lung02 trial (NCT06203210) is underway comparing I-DXd with physician’s treatment of choice in relapsed SCLC. The phase I/II IDeate-Lung03 study (NCT06362252) is set to start recruitment to investigate combinations of I-DXd with atezolizumab with or without carboplatin as first-line induction or maintenance for ES-SCLC.

HS-20093 is another novel B7-H3 targeting ADC with a topoisomerase inhibitor payload being studied for SCLC [63]. In the phase I ARTEMIS-001 trial (NCT05276609) in patients with advanced solid tumors, TRAEs were observed in all 53 patients enrolled though only 3 experienced dose-limiting toxicities. A subset analysis including an expanded SCLC subgroup of 56 patients up from 11 in the prior analysis provided updated expansion dose data [64]. In the group that received 8 mg/kg every 3 weeks during the expansion phase, ORR was 58.1%, disease control rate (DCR) was 80.6%, median DOR was 4.3 months, and median PFS was 5.6 months with a median follow-up time of 4.8 months. In the 10 mg/kg every 3 weeks group, ORR was 57.1% and DCR was 95.2% with a median follow-up time of 4.9 months. Median DOR and PFS were not provided for the 10 mg/kg group. The follow-up phase II ARTEMIS-007 trial (NCT06052423) in ES-SCLC has been withdrawn due to a research and development strategy adjustment though HS-20093 is still under investigation in two trials involving SCLC: the phase III ARTEMIS-008 study (NCT06498479) comparing HS-20093 to topotecan in relapsed SCLC and the phase III study (NCT06526624) comparing HS-20093 to active surveillance in LS-SCLC.

### Seizure-related homolog 6 (SEZ6)

Seizure-related homolog 6 (SEZ6) is a novel SCLC target identified using mRNA expression analysis and IHC. It was described to be highly expressed on the surface of SCLC cells with minimal expression on normal tissues [65].

ABBV-011 is a SEZ6 antibody SC17 paired with the calicheamicin (topoisomerase I inhibitor) linker drug LD19.10. Preclinical trials demonstrated promising antitumor activity. A phase I trial (NCT03639194) aimed at investigating safety and tolerability of ABBV-011 monotherapy or paired with budigalimab, a programmed cell death 1 inhibitor was performed in participants with relapsed or refractory SCLC though data from the dual therapy cohort has not been published [66]. For the ABBV-011 monotherapy cohort, maximum tolerated dose (MTD) was not reached while ORR was 25% and median PFS was 3.5 months. Grade 3 or greater TRAEs occurred in 47.5% of patients, the most common being fatigue, thrombocytopenia, and neutropenia at 10% each. While this trial demonstrated the tolerability and promising efficacy of ABBV-011, further trials have not been initiated at this time.

ABBV-706 is another anti-SEZ6 ADC that utilizes a novel topoisomerase 1 inhibitor payload. A phase I trial (NCT05599984) in various advanced solid tumors showed ABBV-706 was well tolerated with grade 3 or greater TRAEs in 57% and the most common were neutropenia (29%), anemia (27%), and leukopenia (25%) [67]. ORR was 21% overall and 40% in the SCLC group. This trial is ongoing with additional cohorts receiving ABBV-706 in combination with budigalimab, cisplatin, or carboplatin in patients with advanced solid tumors.

### Heat shock protein 90 (Hsp90)

Heat shock protein 90 (Hsp90) is a molecular chaperone protein that has long been a target for antitumor therapies. It was found to be integral to suppressing apoptosis in SCLC leading to the development of anti-Hsp90 therapies [68].

PEN-866, formerly known as STA-8666, is an anti-Hsp90 ADC composed of an anti-Hsp90 mAb, cleavable carbamate linker, and SN-38 payload. Preclinical studies demonstrated the efficacy of PEN-866 in SCLC tissue models [69]. A phase I study (NCT03221400) in advanced solid malignancies including SCLC demonstrated adequate tolerability and promising antitumor activity with stable disease in 11 patients and decreased target lesion size in 6 additional patients of the 26 evaluable patients at the end of the study [70]. A phase 2 expansion study was planned but was suspended for an unspecified reason.

### ERBB family

The ERBB family are receptor tyrosine kinases that have been implicated in tumor initiation and proliferation. This family includes EGFR (also known as ERBB1 and HER1) and EGFR3 (also known as ERBB3 and HER3). HER2 is a member of the ERBB family that is expressed in up to 10% of SCLC and associated with worse prognosis [71]. Due to this association with poor outcomes and strong expression in solid tumors including SCLC and NSCLC, targeted therapies against ERBB family receptors have been heavily investigated [72].

BL-B01D1, a first-in-class EGFR-HER3 bispecific ADC, contains a bispecific antibody composed of an anti-EGFR human IgG1 antibody linked to two anti-HER3 human single-chain fragment variables (scFvs) by a glycine-serine linker, a tetrapeptide-based cleavable linker, and Ed-04 payload which is a camptothecin (topoisomerase I inhibitor) derivative [72]. Preclinical studies demonstrated increased anti-tumor activity for this bispecific ADC compared to ADCs targeting either EGFR or HER3 with identical payload and linkers suggesting there is benefit to utilizing this bispecific design [73]. BL-B01D1 was investigated in a phase I trial (NCT05194982) in patients with various locally advanced or metastatic solid tumors. Overall, there were 195 patients enrolled and amongst this group, 71% experienced grade 3 or greater TRAEs and 36% had serious TRAEs [72]. The most common grade 3 or greater TRAEs were neutropenia (47%), anemia (39%), leukopenia (39%), and thrombocytopenia (32%). 27% of participants required dose reductions due to TRAEs and 3% discontinued treatment altogether. 174 of the enrolled patients were evaluable for antitumor activity and results showed an overall partial response rate of 34%, ORR of 34%, median DOR of 8.5 months, median PFS of 5.7 months, and disease control in 89% of participants. 13 patients with SCLC were enrolled in this study but subgroup analysis was not included. BL-B01D1 is a promising new ADC and there are several active trials for its application in SCLC and solid tumors in general (Table 2).

### Carcinoembryonic antigen-related cell adhesion molecule 5 (CEACAM5 or CD66e)

Carcinoembryonic antigen-related cell adhesion molecule 5 (CEACAM5) was an early target of immunotherapeutic investigation and was of interest due to its role in adhesion and invasion of tumor cells [74]. In vitro studies demonstrated the utility of targeting CEACAM5 as mAbs against the antigen led to increased survival in mouse models.

Tusamitamab ravtansine (SAR408701) is an anti-CEACAM5 ADC composed of a humanized mAb against the extracellular domain of CEACAM5, a cleavable disulfide linker, and ravatasine (DM4), a maytansinoid payload [75]. A phase I trial (NCT02187848) in patients with advanced solid tumors assessed safety and toxicity through a two-phase dose escalation study. In the first phase, the MTD was determined to be 100 mg/m^2^ every 2 weeks (Q2W). In the second phase, two cohorts were created: the Q2W-LD cohort which received escalating loading doses of tusamitamab ravtansine on day 1, cycle 1 followed by the MTD administered Q2W and the Q3W cohort which received escalating doses of tusamitamab ravtansine administered every 3 weeks. In terms of toxicity, 4 of 28 (14.3%) patients in the Q2W-LD cohort had grade 3 or higher TRAEs, which were keratopathy, keratitis, and decreased platelet count. 2 of 15 (13.3%) patients in the Q3W cohort had grade 3 or greater TRAEs, which were comprised of keratopathy and elevated transaminases. There were no recorded serious TRAEs for the Q2W-LD cohort though one of 6 (16.7%) patients at the 170 mg/m^2^ dose level in the Q3W cohort had a serious TRAE of grade 2 drug infusion-related reaction during cycle 1. There was an expansion cohort of patients with CEACAM5 expressing SCLC that was terminated by the sponsor of the study and was not related to any safety concerns.

## BiTEs in SCLC

BiTEs are a promising development for cancer therapy. BiTEs are bispecific antibodies composed of two scFvs, which are the variable domains of the Fab region of antibodies [76]. BiTEs achieve bispecificity through pairing a scFv targeted at tumor-associated antigen and a scFv directed at CD3 on T cells [77]. This bispecificity allows for the identification of tumor cells and recruitment of T cells directly to these targeted cells, maximizing the potential of T cells in tumor regulation and overcoming the evasion processes of tumor cells.

Studies in leukemia and lymphoma showed promise in the practical application of BiTEs. Blinatumomab is the first FDA-approved BiTE and has now been approved for three indications: consolidation phase of CD19-positive Philadelphia chromosome-negative B-cell precursor acute lymphoblastic leukemia (BCP-ALL), MRD-positive CD19-positive BCP-ALL in first or second complete remission, and in relapsed or refractory CD19-positive BCP-ALL.

Development of BiTEs for solid tumors has shown to be challenging with toxicities and development of resistance contributing to the failure of early trials, but through these challenges, much has been learned about possible routes of optimization for BiTE therapy. Catumaxomab was an example of this; it was a bi-specific (anti-EpCAM & anti-CD3) trifunctional (FCγ receptors) antibody that was shown to be effective in peritoneal carcinomatosis but trials in systemic administration demonstrated immune-mediated hepatotoxicity [78]. ​It was determined that the Fc region of catumaxomab was able to bind the FCγ receptor of Kupffer cells leading to the observed off-target effects. Since then, BiTEs have been largely designed without Fc domains altogether or at least contain Fc domains with reduced FCγ reactivity [79]. The MEDI-565 trial in advanced gastrointestinal adenocarcinomas demonstrated another limitation, as it induced the production of antidrug antibodies [80]. Since the results of this trial, the immunogenicity of BiTEs has been limited through the utilization of humanized scFvs [79]. Recently, SCLC has been the stage for discovery and advancement of BiTE therapy in hopes of overcoming these challenges. Tarlatamab has been the focal point and is the only BiTE that has been investigated in SCLC with two completed trials (Table 4) and several active and recruiting.

**Table 4:** Key trials of BiTEs in SCLC

**Agent**	**Tumor target/T cell target**	**Trial identifier**	**Phase**	**ORR**	**OS**	**PFS**	**Grade 3 or > TRAEs**
Tarlatamab	DLL3/CD3	DeLLphi-300 (NCT03319940)	I	23.4% (95% CI: 15.7–32.5)	13.2 months (95% CI: 10.5–not reached)	3.7 months (95% CI: 2.1–5.4)	
Tarlatamab	DLL3/CD3	DeLLphi-301 (NCT05060016)	II	40% (97.5% CI: 29–52) 10 mg, 32% (97.5% CI: 21–44) 100 mg		4.9 months (95% CI: 2.9–6.7) 10 mg, 3.9 months (95% CI: 2.6–4.4) 100 mg	26% 10 mg, 33% 100 mg

BiTEs: bispecific T-cell engagers; SCLC: small cell lung cancer; DLL3: delta-like ligand 3; CI: confidence interval; ORR: objective response rate; OS: overall survival; PFS: progression-free survival; TRAEs: treatment-related adverse events

### DLL3

DLL3 thus far is the most studied target of BiTE therapies in solid tumors with most therapies in investigation specifically for SCLC.

Tarlatamab, formerly AMG 757, is a half-life extended (HLE) BiTE composed of an anti-DLL3 scFv, an anti-CD3 scFv, a short, flexible linker, and a stable, effector-functionless IgG Fc domain which serves to increase half-life of the molecule. The shorter half-life of canonical BiTEs is largely attributed to the small size of these molecules as they are composed of only two scFv regions making BiTEs susceptible to rapid renal clearance [76]. This required continuous IV infusion to maintain therapeutic concentrations, but HLE BiTEs like tarlatamab have improved half-life by increasing molecule size through the incorporation of an IgG Fc domain. Increased toxicity was naturally a concern for HLE BiTEs due to their reduced clearance, but a preclinical study in nonhuman primates comparing a canonical anti-DLL3 BiTE to tarlatamab demonstrated an extended half-life for tarlatamab without increased toxicity [76, 81]. Overall, preclinical studies showed promising results for efficacy and safety of tarlatamab, supporting its study in human subjects.

The phase I DeLLphi-300 trial (NCT03319940) in patients with relapsed or refractory SCLC was primarily aimed at investigating safety of tarlatamab at multiple doses with secondary results in antitumor activity, survival, and pharmacokinetics [82]. Tarlatamab was found to have manageable tolerability through all studied doses in the dose escalation phase which tested doses from 0.003 mg to 100 mg via IV infusion Q2W. The most common TRAE was cytokine release syndrome (CRS) which occurred in 56% of study participants though symptoms were overall short-lived, reversible, and mostly during the first cycle of treatment. ORR was 23.4% (95% CI: 15.7–32.5) with 2 complete and 23 partial responses, the median duration of response was 12.3 months (95% CI: 6.6–14.9). The median PFS was 3.7 months (95% CI: 2.1–5.4) and the median OS was 13.2 months (95% CI: 10.5–not reached). An exploratory post hoc analysis found clinical benefit in patients with higher total DLL3 expression.

The phase II DeLLphi-301 trial (NCT05060016) in patients with previously treated SCLC provided exciting data about the antitumor activity and safety of tarlatamab [83]. Patients received tarlatamab at doses of 10 mg and 100 mg by IV administration Q2W. ORR was 40% (97.5% CI: 29–52) for the 10 mg dose group and 32% (97.5% CI: 21–44) for the 100 mg group. Among the participants with OR, ongoing OR was observed at time of data cutoff in 55% of the 10 mg group and 57% in the 100 mg group. DOR was at least 6 months in 59% of patients who had OR in both groups. Median PFS was 4.9 months (95% CI: 2.9–6.7) for the 10 mg group and 3.9 (95% CI: 2.6–4.4) in the 100 mg group. OS at 6 months was 73% in the 10 mg group and 71% in the 100 mg group. OS at 9 months was 68% for the 10 mg group and 66% for the 100 mg group. CRS was again the most common TRAE and was observed in 51% of the 10 mg group and 61% of the 100 mg group. Grade 3 or higher TRAEs occurred in 26% and 33% of the 10 mg and 100 mg groups respectively with treatment discontinuation in 3% of each group. A single patient in the 10 mg group died from respiratory failure which was determined to be related to the trial therapy. From the results of this trial, the 10 mg dose was determined to have the most favorable benefit-to-risk profile and selected to be the dose for subsequent trials. In terms of DLL3 expression, 83% of the 157 patients in the study had testable tissue samples and 96% of the samples were positive for DLL3.

There are several trials investigating tarlatamab combination therapies. DeLLphi-302 (NCT04885998) is a phase I trial that is underway investigating tarlatamab in combination with anti-PD-1 therapy in second-line or later SCLC. DeLLphi-303 (NCT05361395) is a second phase I study of tarlatamab in combination with standard of care therapies in first-line SCLC. The DeLLphi-304 (NCT05740566) is a phase III trial investigating tarlatamab in combination with standard of care therapy for patients with relapsed SCLC after first-line platinum-based chemotherapy.

The phase III DeLLphi-305 study (NCT06211036) is investigating tarlatamab for first-line treatment in ES-SCLC. This trial is currently recruiting participants to compare tarlatamab in combination with durvalumab to durvalumab alone after platinum, etoposide and durvalumab treatment. The phase III DeLLphi-306 study (NCT06117774) is also recruiting and investigating the application of tarlatamab in LS-SCLC after chemoradiotherapy.

Due to the results of the early DeLLphi trials, tarlatamab became the first BiTE therapy to be FDA-approved for the treatment of any solid tumor. Several other studies are now looking to explore further applications of tarlatamab and other DLL3-targeted BiTEs including BI 764532, HPN328, RO7616789, PT217, and QLS31904.

## CAR-T cell therapies in SCLC

In addition to ADCs and BiTE therapies, adoptive cell transfer therapies such as CAR-T cells, tumor-infiltrating lymphocytes, TCR-engineered T cells, and CAR-modified NK cells, are being explored as advanced approaches to overcome the limitations of conventional therapies. CARs are engineered receptors composed of an extracellular scFv that functions as the targeting moiety, a transmembrane spacer, and an intracellular activation domain. These constructs are introduced into T cells allowing them to target surface-exposed tumor-associated antigens [84]. The evolution of CAR structure has progressed through several generations. First-generation CARs featured the CD3ζ signaling domain, providing basic activation signals. Second-generation CARs incorporated an additional costimulatory signaling domain such as 4-1BB, CD28, CD27, ICOS, or RIAD to enhance T cell activation and persistence. Third and fourth generation CARs now feature two or more signaling domains to further augment T cell persistence and proliferation [85]. At time of this review, validation of the CAR-T platform has been achieved with six CAR-T products engineered to express receptors for either CD19 or B-cell maturation antigen, approved by the FDA for the treatment of various hematologic malignancies [86, 87].

Despite the success of CAR-T cell therapy in hematologic malignancies, its application to solid tumors, including SCLC, has faced significant challenges [88]. The TME in solid tumors presents a formidable obstacle, often with an immunosuppressive milieu containing regulatory T cells, myeloid-derived suppressor cells, and other factors that inhibit CAR-T cell activity [89]. Additionally, dense extracellular matrix and abnormal tumor vasculature can impede CAR-T cell infiltration into the tumor [90]. T cell exhaustion and short persistence is another critical issue, where CAR-T cells become functionally impaired due to chronic antigen exposure and the immunosuppressive TME, leading to reduced efficacy [89]. In most solid tumor trials [91], CAR-T transcripts remain detectable for about a month following infusion as compared to CD19-targeted CAR-T cells with persistence ranging up to years [87]. Antigen heterogeneity in solid tumors, including SCLC, poses another challenge, with antigen escape leading to reduced CAR-T cell efficacy [92] and safety concerns are of concern with the risk of on-target, off-tumor toxicity when targeting antigens are also expressed on normal tissues.

**Figure 4:**
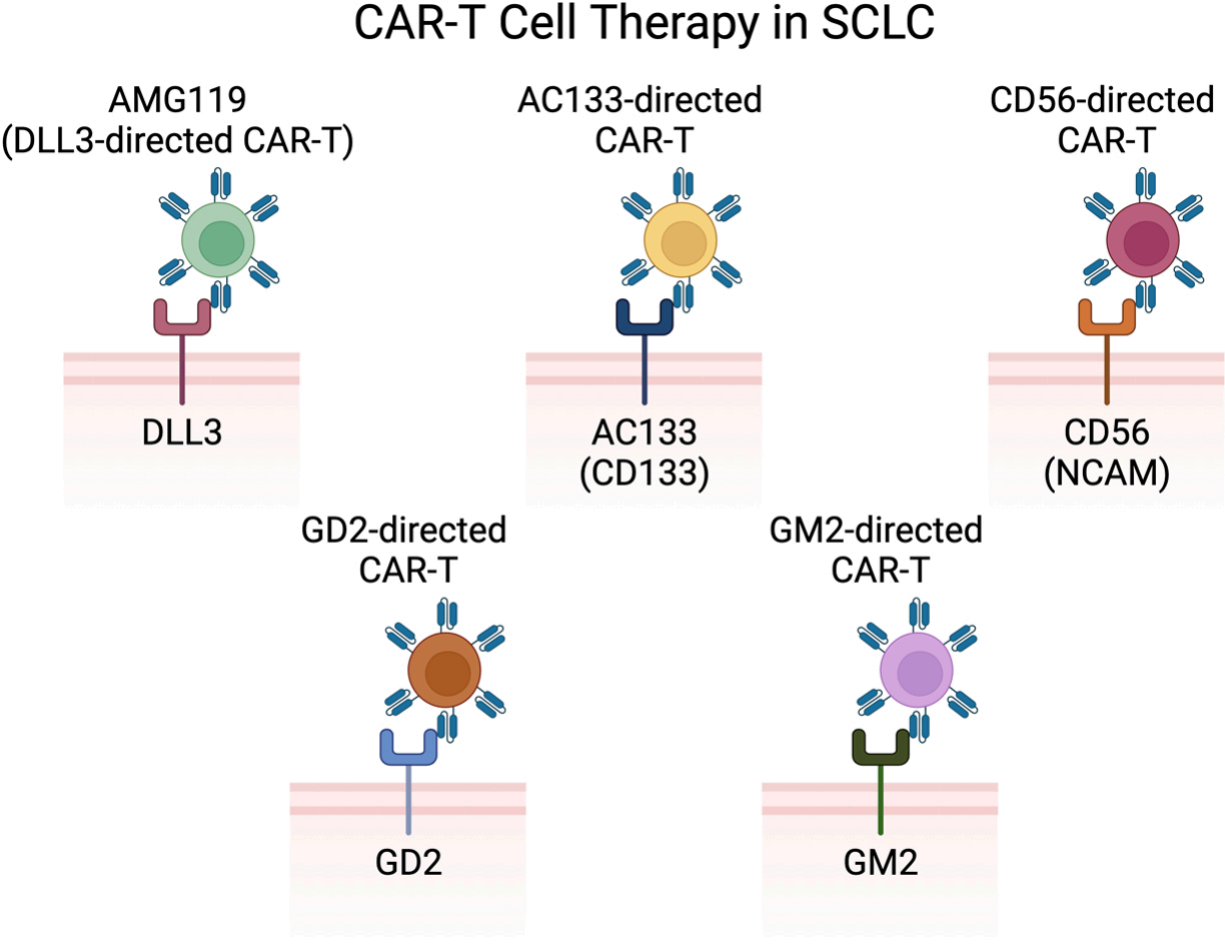
**CAR-T cell therapies under investigation in SCLC include anti-DLL3 CAR-T, anti-AC133 CAR-T, anti-CD56 CAR-T, anti-GD2 CAR-T, and anti-GM2 CAR-T** [38]. CAR: chimeric antigen receptor; SCLC: small cell lung cancer; DLL3: delta-like ligand 3; GD2: gangliosides disialoganglioside GD2; GM2: ganglioside GM2. Created in BioRender. Poei, D. (2025) https://BioRender.com/f38i703

There are four active trials including patients with SCLC investigating CAR-T cell therapies (Figure 4 and Table 5): anti-DLL3 NK cells, anti-DLL3 CAR-T cells, and anti-gangliosides disialoganglioside GD2 (GD2) and iC9 CAR-T cells.

**Table 5:** Trials of CAR-T cell therapies in SCLC

**Cell type**	**Agent**	**Phase**	**Status**	**Trial identifier**	**Indications**	**Sponsor**
NK cells	Anti-DLL3 NK cells	I	Recruiting	NCT05507593	R/R ES-SCLC	Tianjin Medical University
T cells	Anti-DLL3 CAR-T cells	I	Recruiting	NCT05680922	R/R ES-SCLC, LCNEC	Legend Biotech
T cells	Anti-DLL3 CAR-T cells (SNC115)	I	Recruiting	NCT06384482	R/R ES-SCLC, LCNEC	Shanghai Simnova Biotech
T cells	Anti-DLL3 CAR-T cells (AMG 119)	I	Suspended	NCT03392064	R/R ES-SCLC	Amgen
T cells	Anti-GD2 and iC9 CAR-T cells (iC9.GD2.CAR.IL-15 T cells)	I	Recruiting	NCT05620342	R/R ES-SCLC, stage IV NSCLC	UNC Lineberger
T cells	Anti-PD-L1/4-1BB DLL3 CAR-T cells (BHP01)	I	Not yet recruiting	NCT06348797	R/R ES-SCLC	Sichuan University

CAR: chimeric antigen receptor; SCLC: small cell lung cancer; DLL3: delta-like ligand 3; GD2: gangliosides disialoganglioside GD2; PD-L1: programmed death-ligand 1; ES-SCLC: extensive-stage SCLC; NSCLC: non-SCLC

### DLL3

**Figure 5:**
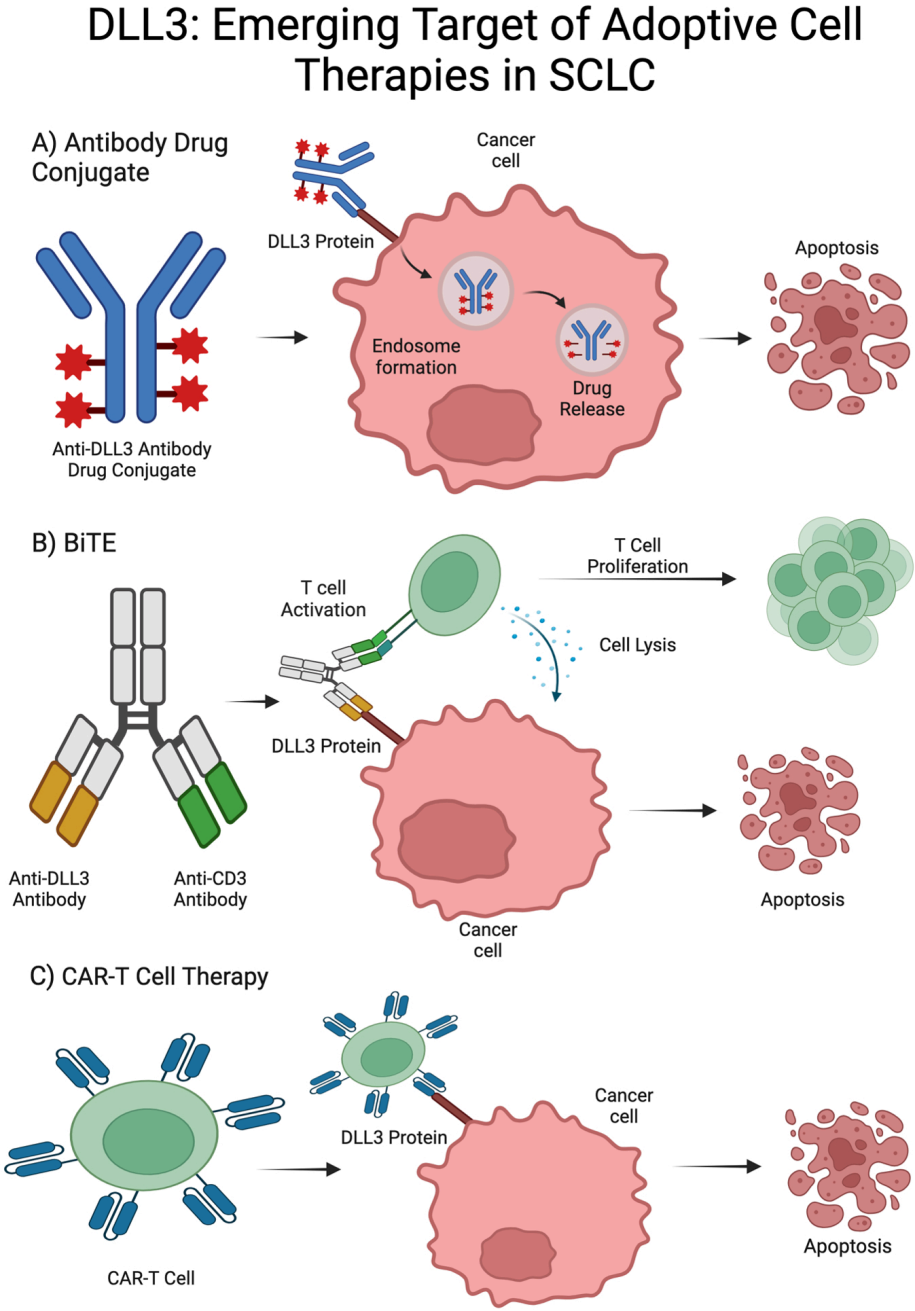
**ADC therapeutic subclasses targeting DLL3 and mechanisms of action**. (**A**) Anti-DLL3 ADCs investigated in SCLC include rovalpituzumab tesirine and FZ-AD005; (**B**) anti-DLL3 BiTEs investigated in SCLC include tarlatamab, BI 764532, HPN328, RO7616789, PT217, and QLS31904; (**C**) anti-DLL3 CAR-T therapies investigated in SCLC include AMG119 and SNC115 [38]. DLL3: delta-like ligand 3; SCLC: small cell lung cancer; BiTEs: bispecific T-cell engagers; CAR: chimeric antigen receptor; ADC: antibody drug conjugate. Created in BioRender. Poei, D. (2025) https://BioRender.com/l72u654

Similar to its investigations with ADCs and BiTEs, DLL3 has emerged as a promising target for CAR-T cell therapy (Figure 5) due to its substantial over-expression in SCLC and the absence of cell surface DLL3 in non-malignant cells [93]. Several studies and clinical trials have focused on developing DLL3-targeted CAR-T cells. AMG119 represents a third-generation CAR-T expressing CD28 and 4-1BB and an anti-DLL3 binding domain. In a phase I clinical trial (NCT03392064) of relapsed/refractory SCLC, AMG119 elicited tumor responses in 1 patient and stable disease in another, including a complete response in hepatic metastasis [94]. Despite these results, the trial was suspended in 2021 after enrolling just five patients with reasons not disclosed. Other in-human trials are currently underway, including a phase I trial (NCT05680922) studying LB2102 in ES-SCLC, a phase I trial studying SNC-115 in recurrent/refractory SCLC and neuroendocrine carcinoma (NCT06384482), and a phase I trial investigating BHP01, a DLL3 targeted α-PD-L1/4-1BB CAR-T (NCT06348797). Further studies involving ALLO-213, an allogeneic DLL3 CAR-T chosen from a selection of scFv-based anti-DLL3 candidates in a subcutaneous tumor murine model, are also currently underway [95].

In recent years, novel CAR structure designs have also been used in preclinical models to enhance CAR-T cells’ resistance to the solid TME. A preclinical study by Jaspers et al. [96] utilized IL-18 secreting “armored” DLL3 targeting CAR-T cells. In this orthotopic and metastatic model, the addition of T-cell IL-18 secretion demonstrated enhanced anti-tumor activity compared to traditional DLL3-targeting CAR-T alone, with improved T cell proliferation, persistence, and reduction in T cell exhaustion markers. Upregulation of PD-L1 on tumor cells and myeloid effector cells was also seen with improved CAR-T cell response with delayed immune checkpoint blockade. Another significant advancement is multiple chain CAR-T designed by Nie et al. [97], which targets DLL3 using the TREM1 receptor and DAP12 adapter protein. This approach demonstrated superior anti-tumor efficacy in a xenograft model compared to second-generation CAR-T cells. The TREM1/DAP12 system leverages DAP12 as a signaling module, enhancing CAR-T cell activation and function through pathways common in myeloid cell signaling. This design offers a promising avenue for overcoming the challenges posed by the immunosuppressive environment of solid tumors such as SCLC.

Complementing CAR-T cell therapy, preclinical studies of DLL3 CAR-NK cells, specifically using the NK-92 cell line equipped with the NKG2D transmembrane domain and co-stimulatory 2B4-CD3 domain, showed significant antitumor activity in SCLC subcutaneous xenograft models and pulmonary metastasis models [98]. This configuration potentially enhances the cytotoxic effects of NK cells, offering a promising avenue in cancer therapy and advantages such as “off-the-shelf” availability [99]. DLL3 CAR-NK are now being studied in a phase I clinical trial (NCT05507593).

### AC133

AC133, also known as CD133, is a transmembrane glycoprotein identified as a cancer stem cell marker linked to several metastatic malignancies and tumor relapse. In a recent study, a single dose of AC133-directed CAR-T induced histologic remission in mice xenografted with brain metastasis cells from a primary lung source [100], though mice in this model eventually relapsed and succumbed to their tumor burden. AC133 CAR-T has also been tested in metastatic SCLC models in conjunction with PD-1 inhibition and CD73 inhibition in an orthotopic xenograft mouse model with complete response seen in 25% of mice without evidence of GVHD [101].

### Gangliosides disialoganglioside GD2 (GD2) and ganglioside GM2 (GM2)

The GD2 and ganglioside GM2 (GM2), are also known to be overexpressed on cancer cells and are associated, at least in part, with the malignant properties of cancers, including SCLC [102]. GD2-directed CAR-T has previously demonstrated strong efficacy in neuroblastoma, with a recent phase I/II trial of patients with relapsed/refractory high-risk neuroblastoma treated with third-generation (CD28 + 4-IBB) GD2-specific CAR demonstrating an overall response of 63% and event-free survival of 36% [103]. GD2 is also extracellularly expressed on up to 80% of SCLC tumors [104, 105], with previous studies demonstrating anti-tumor activity of 2G (CD28) CAR-T cells directed against GD2 in xenografted mouse models [106]. A study investigating rejuvenated induced pluripotent stem cell (iPSC)-derived GD2 CAR-T cells demonstrated robust cytotoxicity against SCLC in mice [107]. This promising approach has been recently investigated using iPSCs to generate CAR-T cells capable of overcoming T-cell exhaustion and limited expansion capacity when compared against conventional GD2-CAR-T. Separately, a third-generation CAR-T model against GM2 expressing IL-7 and CCL19 has demonstrated strong efficacy in a SCLC xenograft model [108]. The expression of IL-7 and CCL19 was found to induce significant CAR-T cell tumor infiltration and long term GM2-specific memory responses without detectable adverse events. A current in-human phase I trial of CAR-T cells directed against GD2 and inducible caspase 9 safety switch is currently being studied (NCT05620342).

### CD56

CD56, also known as NCAM (neuronal cell adhesion molecule) is one of the receptors expressed on the surface of neural cells, NK cells, dendritic cells, and a subset of T cells. It is noted that CD56 is often overexpressed in neuroendocrine tumors, including nearly all SCLC types [109]. Lorvotuzumab mertansine, an ADC against CD56, was successful in murine models but did not improve survival in a phase I/II study [110]. Similarly, Crossland et al. [111] demonstrated CD56 directed CAR-T cells in neuroblastoma and SCLC mouse models with significant reduction in tumor burden after infusion, and a modest effect on OS, though safety concerns remain due to the potential for on-target, off tissue toxicity, particularly given CD56’s expression in neural cells.

## Discussion and future directions

Despite improvements with ICIs in SCLC, only approximately 10% of patients experience a durable response to chemotherapy and ICIs [3]. Efforts to define the immune pathway and identify consistent biomarkers for response or survival in SCLC have been challenging; traditional immunotherapy markers, such as TMB and PD-L1, have not been shown to be a reliable predictor of immunotherapy response in SCLC. The high degree of inter and intra tumoral heterogeneity and neuroendocrine differentiation in SCLC has also made it difficult to identify universally applicable biomarkers and develop personalized therapies. While loss of function mutations in *TP53* and *RB1* are near-universally expressed in SCLC, they remain non-actionable drug targets. Other recurrent mutations—such as MYC alterations, the *NOTCH* gene family, *PTEN* loss, *PI3K* activation, and *FGFR1* amplification-are yet to be validated as therapeutic targets or predictive markers in SCLC [112].

Conflicting data from other malignancies further complicate biomarker development; for instance, although Trop2 overexpression is associated with improved responses in advanced TNBC, overexpression of Trop2 has not been definitively correlated with improved responses in patients with advanced NSCLC treated with anti-Trop2 ADCs [113]. Similarly, while initial studies showed that SCLC patients with high DLL3 expression (≥ 50% of tumor cells) had better response rates and disease control than those with low DLL3 expression, follow-up studies did not confirm DLL3 overexpression as a predictive biomarker [45]. These inconsistencies highlight the complexity of biomarker development in SCLC and describe the need for more robust, prospective studies to validate potential predictive markers. Despite these challenges, recent studies suggest the presence of distinct molecular SCLC subtypes that may respond differently to therapies, indicating that matching a patient’s baseline subtype to specific treatments could enhance therapeutic response [3]. These findings underscore the need for further research to establish reliable predictive biomarkers.

Adoptive cell therapies have provided new avenues for the treatment of SCLC, and therapeutics targeting DLL3, Trop2, CD276, SEZ6, and GD2 in in-human investigations are currently underway. However, additional research is needed to better understand the toxicities and mechanisms of resistance of adoptive cell therapies as well as expand age-stratified subgroup analysis for these therapies.

ADCs, BiTEs, and CAR-T cell therapies each introduce distinct toxicities that also require careful identification and management that currently limit their broader effectiveness. Frequently, the dose-limiting toxicity (DLT) for most ADCs appear to be off-target effects [39] in addition to the toxicity of the attached payload. ADC regimens have also been associated with fatal toxic events, with respiratory diseases including pneumonitis (12.4% incidence in a pooled analysis) [114] and drug-related interstitial lung disease (26% in trastuzumab deruxtecan in patients with HER2-mutant NSCLC) [115]. With T-cell based therapies such as BiTE and CAR-T, toxicities generally stem from their immunostimulatory mechanism of action, with the most common adverse events including CRS, immune effector cell-associated neurotoxicity syndrome (ICANS), and associated neurologic events [83]. Further, as the use of T-cell based therapies expands, a growing body of research highlights the potential for long-term cardiotoxicity and risk of major adverse cardiovascular events necessitating long-term follow up [116].

With regards to resistance, prior studies have shown that resistance to ICIs often develops in late-stage disease, with the TME, antigen downregulation and overall tumor mutation burden described to be the main modes of resistance [117, 118]. For ADCs, almost all patients with late-stage disease eventually develop resistance to these therapies [119]. Emerging theories regarding the mechanism of resistance include antigen downregulation, drug transporter protein over-expression, defects in ADC trafficking pathways, or alterations in signaling pathways [119]. In BiTE therapies, downregulation or loss of tumor-associated antigens and upregulation of inhibitory immune checkpoints within the TME have been described as major mechanisms of resistance particularly in later stage disease [120, 121]. In CAR-T cell therapy, limited tumor infiltration, antigen loss, T cell exhaustion as well as prior exposure to targeted therapies have led to reduced CAR-T cell efficacy [92, 122]. Identifying inhibitory pathways that impede adoptive cell therapies will be necessary to cover potential escape mechanisms from ACTs. Future studies to address treatment resistance to adoptive cell therapies could include combinatorial approaches or multi-target therapies, as has already been trialed in the case of bispecific ADCs and BL-B01D1, as well as in combination therapy of BiTEs with immune checkpoint inhibition [123]. Further research is needed to identify resistance patterns within current therapies to better predict relapse and improve long-term patient outcomes.

Finally, the real-world application of adoptive cell therapies is limited by the underrepresentation of older adults in clinical trial. Dedicated investigation for this subgroup is of increased important in adoptive cell therapies due to observed physiologic differences in immune system and drug metabolism of older patients which can affect both the efficacy and toxicity of these therapies [124, 125]. For ICIs, studies in patients with advanced solid tumors were found to have disproportionately low representation of older patients [124]. Despite this, pooled analysis of available subgroup data demonstrated OS benefit for older adults. Findings were similar in ADCs with studies having limited data on older adults in both hematologic and solid tumor malignancies though available analysis support that there is likely equivalent efficacy in this subgroup [126]. Age stratified analysis is even more limited in trials of BiTEs and CAR-T therapies though initial studies in hematologic malignancies suggest that efficacy for older patients is similar to the general population [125, 127]. While the available pooled analyses for ADCs, BiTEs, and CAR-T therapies in older adults demonstrate comparable efficacy, the limited amount of data on this subgroup is concerning and must be expanded on to better support the use of these therapies in older populations especially considering the physiologic differences of this group which plays an important role in efficacy and toxicity of adoptive cell therapies.

## Conclusion

Overall, adoptive cell therapies have provided many promising avenues for improving therapeutic agents for patients with SCLC highlighted by the success of tarlatamab. ADCs have been well studied and there are several targets currently under investigation. Despite the abandonment of Rova-T and SG as therapies for SCLC, SG demonstrated clinical benefit in several other malignancies, reinforcing the promise of ADCs in solid tumors. Although there is only a single target being studied in SCLC for BiTEs, this drug class represents one of the most promising options for the future of SCLC therapy. Tarlatamab has inspired further development of DLL3 targeted BiTEs as well as exploration of expanded indications of tarlatamab within SCLC. The application of CAR-T therapy in SCLC has also demonstrated significant growth in recent years. DLL3 and AC133 targeted CAR-T therapies are early in development, though initial results have been promising. There have been several encouraging developments in SCLC therapy which has been historically difficult to improve on. Further research is needed on patient selection, resistance, and toxicity associated with novel adaptive cell therapies in SCLC, including ADC, BiTE, and CAR-T therapies along with biomarker development to improve efficacy and patient selection.

## Abbreviations

ADCs: antibody drug conjugates
ASCL1: achaete-scute homolog 1
BCP-ALL: B-cell precursor acute lymphoblastic leukemia
BiTEs: bispecific T-cell engagers
CAR: chimeric antigen receptor
CDX: cell line-derived xenograft
CEACAM5: carcinoembryonic antigen-related cell adhesion molecule 5
CI: confidence interval
CRS: cytokine release syndrome
Dato-DXd: datopotamab deruxtecan
DCR: disease control rate
DLL3: delta-like ligand 3
EGFR: epidermal growth factor receptor
EP: etoposide plus cisplatin or carboplatin
ES-SCLC: extensive-stage small cell lung cancer
GD2: gangliosides disialoganglioside GD2
GEJ: gastroesophageal junction
GM2: ganglioside GM2
HLE: half-life extended
Hsp90: heat shock protein 90
IC_50_: half-maximal inhibitory concentration
ICANS: immune effector cell-associated neurotoxicity syndrome
ICIs: immune checkpoint inhibitors
I-DXd: ifinatamab deruxtecan
IHC: immunohistochemistry
iPSC: induced pluripotent stem cell
mAb: monoclonal antibody
MRD: minimal residual disease
MTD: maximum tolerated dose
NCAM: neuronal cell adhesion molecule
NEPC: neuroendocrine prostate cancer
NeuroD1: neurogenic differentiation factor 1
NSCLC: non-small cell lung cancer
OR: objective response
ORR: objective response rate
OS: overall survival
PD-1: programmed cell death protein-1
PD-L1: programmed death-ligand 1
PDX: patient-derived xenograft
PFS: progression-free survival
POU2F3: POU class 2 homeobox 3
Q2W: every 2 weeks
Rova-T: rovalpituzumab tesirine
scFvs: single-chain fragment variables
SCLC: small cell lung cancer
SEZ6: seizure-related homolog 6
SG: sacituzumab govitecan
TGI: tumor growth inhibition
TME: tumor microenvironment
TNBC: triple-negative breast cancer
TRAEs: treatment-related adverse events
Trop2: trophoblast cell surface antigen 2

## Declarations

### Copyright

© The Author(s) 2025.
